# The Relations of Water in the Bodily Economy

**Published:** 1889-04

**Authors:** 


### *THE RELATIONS OF WATER IN THE BODILY ECONOMY.*

Dr. J. Harvey Dew read a short paper before the Medical Society of the County of New York, January 28, 1889, relating to the importance of water from a physiological standpoint. He would deal with water as an element necessary to the exercise of certain functions, and in the chemistry of animal life. Both laity and profession showed a sad neglect of certain facts about water, the results of which inattention he saw every day.

The chief of these facts were:

1. The body consisted mainly of water, the latter forming in health from two-thirds to three-quarters of its bulk.

2. Water constituted nearly the total volume of the circulating fluids of the body, the blood, lymph, and digestive secretions.

3. It was the vehicle for the processes of digestion, absorption, and transportation of food.

4. It formed a large part of the ultimate tissues, and must be present in every process of assimilation.

5. It served as a vehicle for the circulation of waste matters.

6. Three or four pints (or pounds) of it were needed daily.

7. It could be observed that all thin persons partook scantily of water, whereas all fleshy people drank freely of it.

8. All diseases involved a disturbance of the processes of digestion, absorption, secretion, and elimination.

9. A large proportion of the discomforts and troubles of the body arose from a poor performance of the functions depending upon water. It was proper that in health two pints of water should leave the body daily by the kidney, two by the skin, and one and a half by the lungs, or five and a half total. Water taken at meal-time was rapidly absorbed or passed through the pylorus. Lack of water caused constipation, made the secretions tenacious, the circulation defective, and the skin dry, and directly caused dyspepsia. The habit of taking water only in the form of such beverages as tea, coffee, and beer, was not a good substitute for the drinking of pure water.—*New York Medical Journal.*

